# Successful palliative peptide receptor radionuclide therapy for impending compression of vena cava due to unresectable liver metastasis of neuroendocrine tumor

**DOI:** 10.17179/excli2019-1220

**Published:** 2019-05-21

**Authors:** Bahar Ataeinia, Christina Loberg, Hanna Kravets, Mohsen Beheshti, Dirk von Mallek, Felix M. Mottaghy, Alexander Heinzel

**Affiliations:** 1 Department of Nuclear Medicine, University Hospital RWTH Aachen, Aachen, Germany; 2 Non-Communicable Diseases Research Center, Endocrinology and Metabolism Population Sciences Institute, Tehran University of Medical Sciences, Tehran, Iran; 3 Department of Radiology and Nuclear Medicine, Maastricht University Medical Center, Maastricht, The Netherlands

**Keywords:** neuroendocrine tumors, metastasis, PRRT, 177Lu-DOTATOC

## Abstract

We present the case of a 77 year old male patient with metastatic pancreatic neuroendocrine tumor (NET). The patient was initially treated by extensive surgical resection that however, led to severe complications with delayed recovery. During follow-up, a number of new liver metastases were detected, one of which was in segment I with impending compression of the inferior vena cava. Due to age and general condition of the patient, instead of further surgical treatment, the patient received four cycles of ^177^Lu-DOTATOC resulting in an overall partial response with successful tumor reduction in liver segment I, resolving an impending compression of vena cava.

## Introduction

Liver metastasis commonly develops in gastroenteropancreatic neuroendocrine tumors (NETs) and affects patients' survival to a high extent. Various symptoms may present due to tumor pressure effect or extensive replacement of normal liver parenchyma (John and Davidson, 2012[2]). Extravascular compression of inferior vena cava (IVC) by tumors is a rare but serious condition that can lead to increased intra-abdominal pressure, thrombosis, lower limb edema, liver and kidney damage and reduced cardiac venous return, causing hemodynamic disturbances and even life threatening hypovolemic shock (Mohammed et al., 2018[3]; Sonin et al., 1992[7]). Proper management of venous obstruction and prevention of potential thrombosis is a vital palliative care besides treating the underlying malignancy, leading to a decline in morbidity and mortality (Friedman et al., 2017[1]). Even though radical surgery of the primary tumor and the liver metastasis is the main curative strategy, masses are not always resectable due to extended size, vast local invasion to critical surrounding structures and patient's underlying conditions.

Herein, we describe a case of moderately differentiated pancreatic NET with progressive liver metastasis and impending IVC compression, 2.5 years after radical surgery. 

## Case Report

A 77 year old male patient was diagnosed in his local hospital with non-functional NET of the pancreatic tail with liver metastasis and malignant vascular and lymphatic invasions in 2014. 

He underwent a resection of the primary pancreatic tumor, accompanied by partial resection of the posterior gastric wall, splenectomy, cholecystectomy and resection of the liver segment IVb. A histological examination demonstrated moderately differentiated NET, stage pT4 pN0 L1, V1, R0, G3 and Ki-67 of 11 %. Multiple postoperative complications, including pancreatic fistula, papillary stenosis, several episodes of pneumonia with pleural effusion, abscess in former spleen site, cardiac arrhythmia and a transient ischemic attack (TIA) led to long-term Intensive care unit admission. 

**Figure 1:**
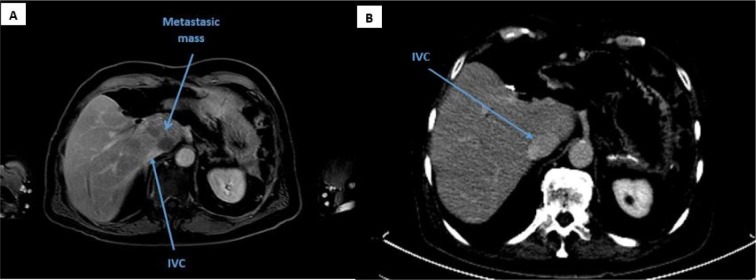
(A): MRI, nine weeks prior to the therapy, revealing the metastatic mass in the liver with an impending IVC compression (IVC diameter: 18.4 x 8.1 mm), (B): CT scan, Twelve months after last PRRT cycle with significant decompression of the vein (IVC diameter: 30.9 x 19.9 mm)

**Figure 2:**
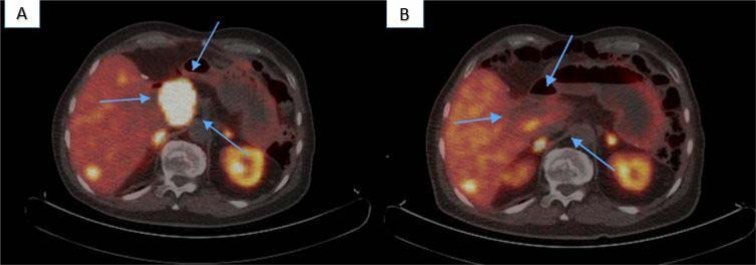
^68^gallium DOTATOC PET/CT; (A): Seven weeks prior to the therapy, revealing intense tracer uptake in the liver lesion (SUV max: 60.78), (B): Twelve months after last PRRT cycle with significant decreased tracer uptake (SUV max: 8.62)

Seven month post-surgery ^68^gallium DOTATOC PET/CT showed a focal tracer uptake in the liver segment I, without corresponding lesion on CT or MRI. Approximately 2.5 years after, ultrasound exam and MRI demonstrated multiple liver metastasis directly adjacent to the IVC (Figure 1A(Fig. 1)). In addition, an intense uptake (SUV max: 60.78) was detected on the ^68^gallium DOTATOC PET/CT, confirming progressive liver metastasis (Figure 2A(Fig. 2)). 

The patient was referred to our hospital for further assessment and treatment. Due to extensive metastasis adjacent to the IVC, high tracer uptake and patient's general condition including history of several post operation complications, cardiac diseases and age, PRRT was recommended by the interdisciplinary tumor board.

The patient was given 4 cycles of ^177^Lu-DOTATOC with cumulative activity of 28.8 GBq, every 2 months. To prevent nephrotoxicity, an amino acid infusion was given in each cycle. No serious side effects were observed during or after the therapy and laboratory data on kidney and bone marrow function were normal. The scintigraphic control performed after the therapies revealed a good uptake to the lesions. 

PET/CT scans 4, 6 and 12 months after the last PRRT cycle revealed further decreasing tracer uptake in liver segment I (SUV max: 7.82) and a very good palliative therapy outcome, in particular with significant reduction of the tumor mass and decompression of the ICV (30.9 x 19.9 mm vs. 18.4 x 8.1 mm) (Figure 2B(Fig. 2)). Even though the patient did not have cholestasis symptoms, a declining pattern toward normal was observed in gamma-glutamyl transferase (223 vs. 99 U/L) and alkaline phosphatase (195 vs. 145 U/L) serum levels, reflecting improvement in biliary tree drainage one year after the therapy.

## Discussion

A significant number of pancreatic NET patients present with locally advanced disease or distant metastasis. Selecting the most appropriate treatment strategy can be a clinical dilemma in some patients. Performing surgery in cases similar to our patient with old age, poor general condition and several post operation complications does not seem to be beneficial (Norton et al., 2011[4]). Current international guidelines recommend using PRRT only as second or third line therapy after failure of first line medical therapies (Pavel et al., 2012[5]). However, in some cases medical treatment may be insufficient for management of extensive tumor in critical locations. Thus, individualized selection of alternative treatment options for such cases seems necessary. In such cases, the current German practice guideline recommends considering ^177^Lu-Dotatate as first-line treatment based on individualized interdisciplinary decision-making by a tumor board (Rinke et al., 2018[6]). 

## Conclusion

We reported successful palliative treatment with ^177^Lu-DOTATOC in a case of pancreatic NET recurrence with extensive unresectable liver metastasis and impending IVC compression. PRRT can be considered as a beneficial treatment strategy in such patients with inoperable extensive masses adjacent to critical vessels.

## Funding

This work was not supported by any specific funding from the public, commercial or not-for-profit sector.

## Conflict of interest

The authors declare that they have no conflict of interest.
